# BCG re-vaccination in Malawi: 30-year follow-up of a large, randomised, double-blind, placebo-controlled trial

**DOI:** 10.1016/S2214-109X(21)00309-0

**Published:** 2021-09-14

**Authors:** Judith R Glynn, Katherine Fielding, Themba Mzembe, Lifted Sichali, Louis Banda, Estelle McLean, Chifundo Kanjala, Amelia C Crampin, Jorg M Ponnighaus, David K Warndorff, Paul E M Fine

**Affiliations:** a Faculty of Epidemiology and Population Health, London School of Hygiene & Tropical Medicine, Keppel Street, London, UK; b Malawi Epidemiology and Intervention Research Unit (formerly Karonga Prevention Study), Chilumba, Malawi; c Pappelweg 6, 08548 Rosenbach (OT Fröbersgrün), Germany; d Kleine Overstraat 23, 7411 JH Deventer, Netherlands

## Abstract

**Background:**

A large, double-blind, randomised, placebo-controlled trial of repeat BCG found 49% efficacy against leprosy but no protection against tuberculosis after 6–9 years’ follow-up in 1995. We report here additional follow-up, which resulted in greatly increased tuberculosis case numbers, and allowed subgroup analysis.

**Methods:**

Nearly 47 000 individuals of all ages living in northern Malawi with a BCG vaccine scar were randomly assigned (1:1) between 1986 and 1989 to receive a second BCG or placebo. The investigators and project staff remained masked to all interventions. Enhanced passive surveillance ensured ascertainment of tuberculosis and leprosy to the end of 2018. Tuberculosis case definitions included rigorous microbiological or histological confirmation. Prespecified subgroup analyses were by tuberculosis type, age at vaccination, time since vaccination, previous tuberculin reactivity, HIV status and *Mycobacterium tuberculosis* lineage. The original trial is registered with ISRCTN registry, ISRCTN11311670.

**Findings:**

In follow-up until Dec 31, 2018, 824 participants had developed tuberculosis, including 786 with pulmonary disease, of whom 383 (63%) of 607 with known HIV status were HIV positive. There was no effect of a second BCG overall (odds ratio [OR] 0·92; 95% CI 0·80–1·05), or for pulmonary (0·93; 0·81–1·07), or lymph node tuberculosis (0·60; 0·31–1·17). The OR was lower for those with known HIV-negative tuberculosis (0·77; 0·59–1·00), for those vaccinated as children (aged <5 years, 0·74; 0·41–1·35; aged 5–14 years, 0·77; 0·60–0·99), and for cases arising at least 20 years after vaccination (0·79; 0·63–1·01). There were no differences by tuberculin status at vaccination, or lineage. There was no evidence of protection against leprosy beyond 10 years after vaccination (although there have been only nine diagnostically certain cases since 1995).

**Interpretation:**

There was no evidence that repeat BCG vaccination provides appreciable protection against overall tuberculosis in this rural African population with a high prevalence of HIV. Subgroup effects should not be overinterpreted given the multiple analyses done. However, the evidence for modest protection against HIV-negative tuberculosis, and for a delayed benefit in those vaccinated as children, is consistent with other observations in the literature.

**Funding:**

LEPRA, Wellcome Trust, Bill & Melinda Gates Foundation.

## Introduction

Repeated BCG vaccination has been policy in many countries in the past, and is still used in several countries, despite lack of good evidence for its effectiveness. WHO's BCG policy guidelines do not recommend it.^1^ However, interest in repeat BCG for protection against tuberculosis has been reignited by a trial in South African adolescents which suggested that BCG revaccination might reduce sustained *Mycobacterium tuberculosis* infection.^2^ Two small studies of BCG revaccination in adults in India and South Africa have found evidence of boosting of T-cell responses.^3^, ^4^ In the absence of alternative strategies, the potential utility of repeat BCG remains an important question. Furthermore, detailed analysis of long-term follow-up after repeat BCG vaccination might reveal patterns important for the development and evaluation of other mycobacterial vaccine options.

To date, there have been two large randomised, controlled trials of repeat BCG, in Brazil and Malawi. In Salvador and Manaus, Brazil, revaccination of schoolchildren aged 7–14 years showed no significant protective effect in an initial analysis, after 5 years of follow-up.^5^ After 9 years, although there was no overall protection, there was evidence of protection in Salvador (an area with low prevalence of environmental mycobacteria exposure) in those vaccinated at age less than 11 years.^6^ No protection was observed against leprosy in this trial.^7^

In Malawi, a large randomised, controlled trial included an evaluation of repeat BCG as well as of vaccines containing BCG plus killed *Mycobacterium leprae*. Analyses after 6–9 years of follow-up revealed that repeat BCG, given irrespective of age and previous tuberculin sensitivity, gave no overall protection against tuberculosis, but 49% protection against leprosy.^8^ At the time of that analysis, in 1995, only 127 participants in the comparison of repeat BCG versus placebo had developed tuberculosis. Now, after 30 years of follow-up, 824 participants have developed tuberculosis in the repeat BCG versus placebo groups of the trial, which allows us to explore patterns by time and to test several hypotheses drawn from the tuberculosis literature.

**Research in context**

**Evidence before this study**

In the absence of a more effective vaccine for tuberculosis there is renewed interest in BCG vaccination and revaccination. Two large trials, in Malawi and Brazil, have reported no overall protection against tuberculosis by repeat BCG. However, a trial in South Africa suggested protection against persistent infection.

**Added value of this study**

This extended follow-up of the Malawi repeat BCG trial greatly increased the power of the study and allowed investigation by subgroup. The lack of overall protection was confirmed, but there were suggestions of delayed protection in those vaccinated as children, among those who were HIV negative, and against extrapulmonary disease. There was no difference in protection by tuberculin sensitivity at baseline, or by *Mycobacterium tuberculosis* lineage.

**Implications of all the available evidence**

The patterns by age and time are consistent with those in Brazil for repeat BCG, and in south India for first BCG. However, any protection was weak, and these results support WHO's policy not to recommend repeat BCG. Development of a more effective vaccine against tuberculosis remains a high priority challenge for research. Patterns revealed in this long-term follow-up should be considered in future evaluations of mycobacterial vaccines.

Experience with primary BCG vaccination led us to expect that vaccine efficacy would be greater in those who were younger at repeat vaccination.^6^, ^9^, ^10^, ^11^ Those vaccinated as young children would have been relatively naive of mycobacterial exposure and in the age range of relatively low tuberculosis incidence at the time of the previous analysis. We expected vaccine efficacy to be higher in those who were tuberculin skin test (TST) negative at repeat vaccination.^2^, ^9^ We collected data on tuberculin reactivity for a subset of the population, but this has not been explored in the trial until now. It has been suggested that vaccine efficacy might vary by *M tuberculosis* lineage:^12^, ^13^ we have genetic sequence data on a high proportion of the *M tuberculosis* isolates, including all four major lineages. We expected greater protection against extrapulmonary than against pulmonary tuberculosis.^9^ We expected greater protection in HIV-negative individuals. In our previous analysis there was some evidence that repeat BCG actually increased the risk for HIV-positive tuberculosis. Numbers were small but it is important to assess this possibility. Vaccine trials for tuberculosis have been restricted to HIV-negative individuals.^2^, ^14^ Although we expected waning vaccine efficacy with time, studies from countries where BCG is protective show evidence of protection for at least 20 years after vaccination.^9^, ^15^

## Methods

### Study design

This was a 30-year follow-up of a large, randomised, double-blind, placebo-controlled trial. The background, design, and methods of the Karonga Prevention Trial in northern Malawi have been described in detail.^16^, ^17^

**Figure 1:**
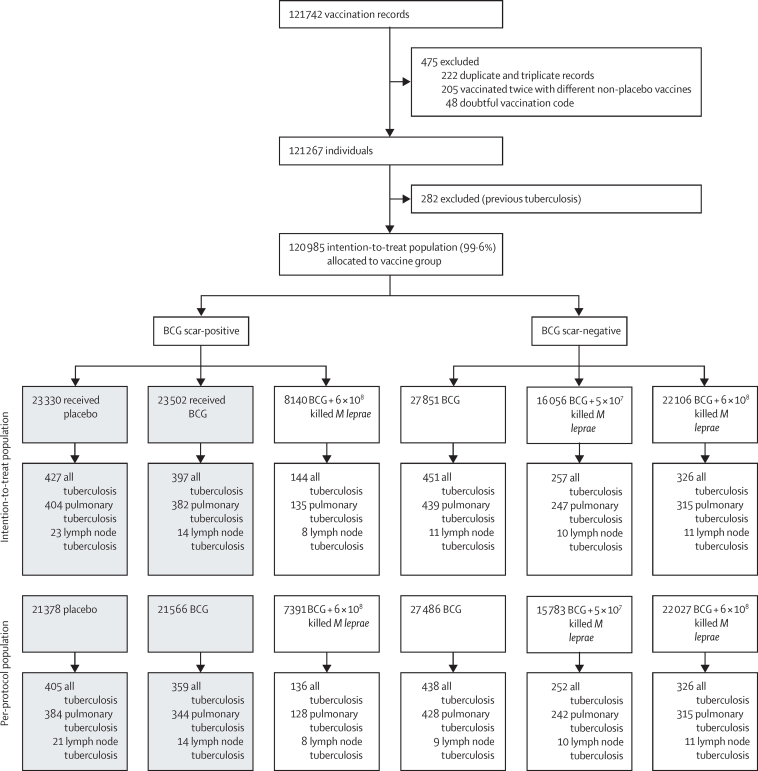
Trial profile The two columns with shaded boxes refer to the repeat BCG versus placebo comparison emphasised in this report. *M leprae=Mycobacterium leprae*.

The trial involved both BCG scar-negative and scar-positive individuals and evaluated vaccination with BCG and with a combined vaccine composed of BCG plus killed *M leprae,* with endpoints of tuberculosis and leprosy. BCG scar-negative individuals received either BCG alone, or BCG plus killed *M leprae* at two different doses. BCG scar-positive individuals received BCG alone, placebo, or BCG plus killed *M leprae* (figure 1)*.* In this report, we concentrate on the effect of repeat BCG, and therefore on the comparison of BCG alone versus placebo in those who were scar-positive at recruitment. We emphasise tuberculosis, since there were few further cases of leprosy after the initial analysis. Additional results on all vaccine combinations, and both diseases, are shown in the appendix. The protocol was approved by the Health Sciences Research Committee of the Malawi Ministry of Health, the Standing Committee on Research in Human Subjects of WHO, and the Ethics Committee of the London School of Hygiene & Tropical Medicine. The trial is registered with the ISRCTN registry, ISRCTN11311670.

### Participants

Virtually the entire population of Karonga District (a small area in the south was excluded), a rural area in northern Malawi, was surveyed between Jan 9, 1986, and Oct 28, 1989, by field teams of trained interviewers, paramedical leprosy control assistants and vaccinators, who visited households systematically. All individuals were questioned about cough, with sputum collection from all reporting a chronic cough or haemoptysis, and were examined for leprosy. Eligible individuals (over 3 months of age and born after 1913, with no evidence of tuberculosis, leprosy, or other serious disease) were invited to participate in the trial, and any reason for exclusion or refusal was recorded. Individuals born before 1914 could request to be included.

### Randomisation and masking

Individuals were randomly assigned to a vaccine depending on their scar status, with individuals with doubtful scar status considered as scar positive. Identical ten-dose vials containing the different trial vaccines were labelled by WHO-appointed trial monitors, packed and used in random order, and unused vaccine in opened vials was destroyed at the end of each day. As ten-dose vials were used, random assignment was by small group rather than by individual: an average of 6·9 (range 1–11) successive scar-negative or scar-positive individuals received vaccine from the same vial. Vaccines were injected intradermally in the right deltoid region.

Vaccine codes were broken by an independent WHO-appointed Data Monitoring Committee (DMC) in 1995, but the investigators and project staff remained masked to all codes. The analysis plan for the extended follow-up, reported here, was discussed and approved by the same independent DMC before codes were broken in 2020.

By agreement with the Malawi Ministry of Health, routine BCG vaccination was suspended in Karonga District during the intake period of the trial. Routine BCG vaccination was then reintroduced, but administered only by project staff, and restricted to infants, to ensure that trial participants did not receive additional doses.

### Procedures

The BCG was Glaxo strain, freeze dried, and the placebo was an identical pellet (the dextran matrix of BCG) both provided by Glaxo. Procedures for production, coding, shipment, storage, and administration of the 11 batches of vaccine used in the trial have been described in detail previously.^16^, ^17^

At the time of recruitment, all participants in selected areas of the district were skin tested with tuberculin (RT23, 2IU).^18^ Skin tests were placed on the volar surface of a forearm (side recorded) and read 48–72 h later, with induration diameters recorded along and across the arm. Average induration diameter was used in all analyses.

Follow-up of the trial population for tuberculosis and leprosy outcomes was by a mixture of active and enhanced passive surveillance, and continued until Dec 31, 2018. The active component included house-to-house surveys in four areas of the district, each with 5000–10 000 population, done in 1991–94. In addition, all individuals known to be first-degree relatives, or to have lived in the household, of a known patient with leprosy since 1980 were traced and examined in 1995. As part of the routine procedures for all studies in the district, including the baseline census for a demographic surveillance site in 2002–04, covering 19 471 (16%) of 121 267 of the trial population, participants were asked about chronic cough, and specimens taken if indicated.^19^, ^20^ All participants living in the demographic surveillance site area have been followed up regularly. In addition, until 2007, project staff were stationed at health centres in the district to screen all attendees for chronic cough and skin lesions.^19^ Since 2007, with the exception of regular follow-up of those in the demographic surveillance population, surveillance has relied on self-reporting to the district health services.

All individuals with suspected tuberculosis or leprosy, regardless of how they were initially detected, were seen by project staff, in conjunction with the National Tuberculosis Programme, and specimens were processed in the project laboratory. Sputum samples were examined by fluorescence microscopy and cultured on acidified Lowenstein-Jensen medium with or without pyruvate. GeneXpert (Cepheid, Sunnyvale, CA, USA) has been available at the district hospital since 2013. Positive cultures with growth macroscopically consistent with mycobacteria were sent to the UK Public Health Laboratory Service–Public Health England Mycobacterial Reference Laboratory for species confirmation, and biopsy specimens were sent to a histopathologist in the UK.^19^ All available *M tuberculosis* isolates up to 2014 were genotyped by means of spoligotyping, restriction fragment length polymorphism, or whole genome sequencing, including stored samples back to 1986.^20^, ^21^, ^22^, ^23^

Individuals diagnosed with tuberculosis and older than 15 years were tested for HIV, after counselling, and if consent was given. HIV serology was carried out by ELISA (HIV-1–HIV-2) and particle agglutination assays and in later years by parallel rapid tests in line with the national testing strategy; serum samples with inconsistent results were tested by additional methods.^24^

### Outcomes

The diagnosis of pulmonary tuberculosis was considered certain if culture, GeneXpert, or genotyping showed *M tuberculosis*, with at least one other specimen positive on culture, GeneXpert, genotyping, or microscopy. The diagnosis was considered probable if culture, GeneXpert, genotyping, or microscopy was positive but not fulfilling the criteria for certainty, and excluding those with only a single scanty sputum smear (ie, fewer than ten bacilli per 100 fields). Smears for which the corresponding culture or genotype showed non-tuberculous mycobacteria were excluded. A diagnosis of extrapulmonary disease was considered certain if the histology was certain or an aspirate was positive on culture, GeneXpert, genotyping, or microscopy (excluding any single scanty smears), and probable if histology was probable or the aspirate had only a scanty positive smear.

Anyone with self-reported or documentary evidence of diagnosed tuberculosis before vaccination was excluded from the analysis for tuberculosis outcomes. Tuberculosis cases arising in the first 6 months (182 days) after vaccination were excluded to ensure onset was post-vaccination. Only the first episode of certain or probable tuberculosis post-vaccination was considered in the analysis.

Diagnostic criteria for leprosy were based on an algorithm incorporating all clinical and histopathological data (see appendix p 17).

### Statistical analysis

The main analysis examined the efficacy of repeat BCG (BCG versus placebo among scar-positives), with diagnostically certain and probable tuberculosis as the endpoint. This included all tuberculosis cases and then considered pulmonary and extrapulmonary tuberculosis separately. We prespecified subgroup analyses for pulmonary tuberculosis, by TST status at vaccination (<5 mm, 5–16 mm, ≥17 mm), by age at vaccination (<5 years, 5–14 years, 15–24 years, ≥25 years), and by time since vaccination (<10 years, 10–19 years, ≥20 years). We also prespecified analyses by HIV status at diagnosis (negative, positive and on antiretrovirals for <3 months, positive and on antiretrovirals for ≥3 months) and (for all tuberculosis) by *M tuberculosis* lineage.

In the 1995 analysis, incidence rates were calculated with person-years denominators. Given the length of the follow-up, more than half the trial population are now likely to have died or left the district, so incidence rates cannot be used. However, follow-up by vaccine group should not be biased. This was tested formally for that portion of the trial population in the demographic surveillance area and showed no evidence of differential mortality or emigration between vaccine groups (appendix p 3). The analyses therefore use logistic regression, adjusting for vaccine batch (where possible), and we report odds ratios (ORs) with their associated 95% CIs, and two-sided p values, from the likelihood ratio test. No adjustment was made for multiple comparisons. For the age at vaccination and TST status subgroups, stratum-specific adjusted ORs for study group are reported, alongside the p value for interaction. Because HIV status is only known for the cases, and *M tuberculosis* lineage and time to diagnosis are only defined for the cases, the overall denominator in each study group is used, and interaction cannot be tested. This assumes that the population HIV prevalence and the duration of follow-up are balanced by study group.

On recommendation of the DMC, the main analysis used an intention-to-treat approach, with individuals analysed according to the vaccine allocated to them. In addition, a per-protocol analysis was run excluding individuals with doubtful or missing vaccine scar status, and any who were allocated a vaccine inconsistent with their recorded scar status. The key comparisons were also re-run restricted to certain tuberculosis as the outcome. These analyses and those for evaluation of the BCG plus killed *M leprae* vaccines are presented in detail in the appendix.

### Role of the funding source

The funders of the study had no role in study design, data collection, data analysis, data interpretation, or writing of the report.

## Results

During recruitment, 5757 residents were found not to be eligible for the trial, and 5835 refused to participate.^8^ As shown in the CONSORT flow diagram (figure 1), 120 985 individuals recruited between Jan 9, 1986, and Oct 28, 1989, contributed data to the 30-year follow-up for tuberculosis outcomes. This excludes 48 individuals with invalid vaccine codes, and 205 who were vaccinated more than once with different non-placebo vaccines. In addition, 282 individuals who had had previous tuberculosis were excluded from the tuberculosis analyses (and 64 individuals with previous evidence of leprosy were excluded from the leprosy analyses, as shown in the appendix p 18).

Among scar-positive individuals, 23 502 were randomly assigned to receive BCG and 23 330 to receive placebo (figure 1). The distributions by study group for age at vaccination, sex, and TST status at vaccination are shown in the appendix (p 7). Among these individuals, 824 developed certain or probable tuberculosis, of whom 786 had pulmonary disease. Almost all diagnosed tuberculosis occurred in adults, and 383 (63%) of 607 participants with pulmonary tuberculosis and known HIV status were HIV positive (appendix p 6).

**Figure 2:**
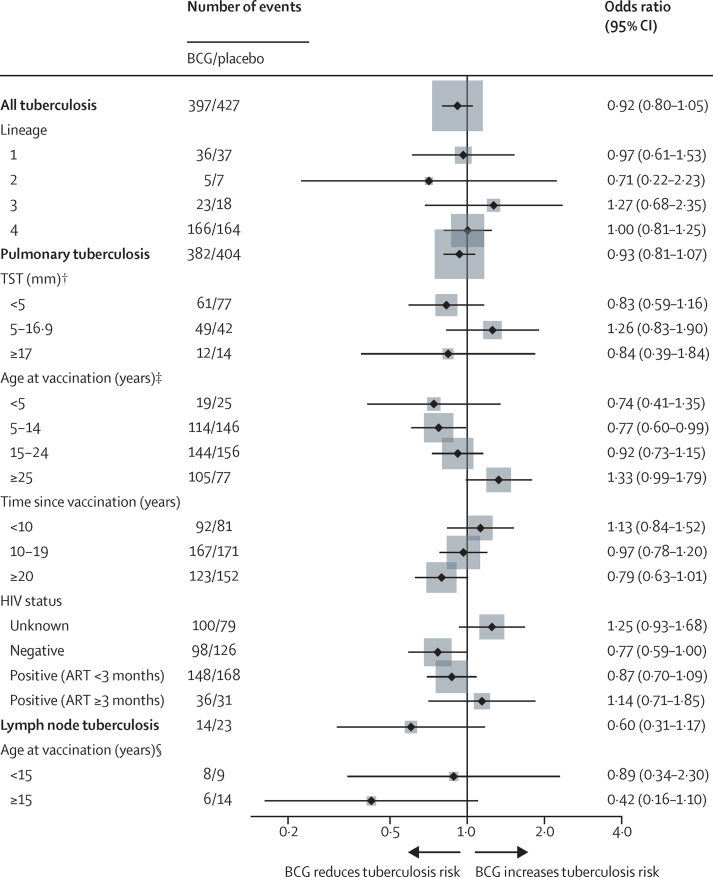
Odds ratios of tuberculosis associated with repeated BCG among scar-positive individuals allocated either repeat BCG or placebo (intention-to-treat population for certain and probable tuberculosis) TST=tuberculin skin test. ART=antiretroviral therapy. Box areas are proportional to sample size. Subgroup analyses are for pulmonary tuberculosis, except lineage, which is based on all tuberculosis. † Interaction p value=0·30. ‡Interaction p value=0·040. §Interaction p value=0·28.

The effect of repeat BCG on tuberculosis is summarised in figure 2. The OR for all certain and probable tuberculosis was 0·92 (95% CI 0·80–1·05). It was 0·93 (0·81–1·07) for pulmonary tuberculosis alone, and 0·60 (0·31–1·17) for lymph node tuberculosis. Only one patient had extrapulmonary tuberculosis that did not involve lymph nodes. For pulmonary disease there was weak evidence of protection in those vaccinated as children, but not for adults (p=0·040 for interaction). There was no evidence of protection in the first 20 years since vaccination, but weak evidence for the period 20–30 years after vaccination. There was no evidence of protection for HIV-positive tuberculosis, but for HIV-negative tuberculosis the OR was 0·77 (0·59–1·00). The OR was lower for those vaccinated as children (aged <5 years, 0·74; 0·41–1·35; aged 5–14 years, 0·77; 0·60–0·99), and for cases arising at least 20 years after vaccination (0·79; 0·63–1·01). There was no evidence of differential protection by previous TST status or by *M tuberculosis* lineage.

**Figure 3:**
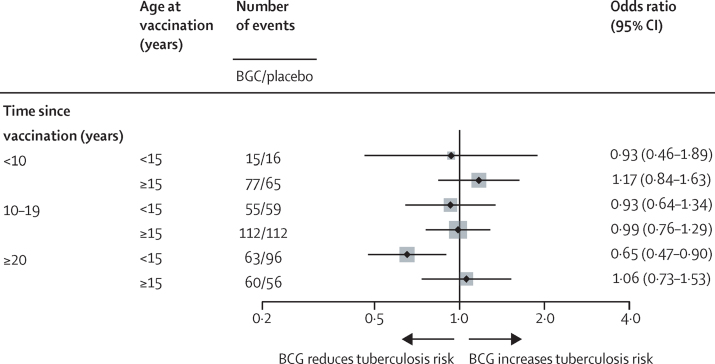
BCG *vs* placebo in scar-positive individuals, stratified by age and years since vaccination (intention-to-treat population for certain and probable pulmonary tuberculosis) p value for interaction between age group and study group by years since vaccination: <10 years, p=0·57; 10–19 years, p=0·79; ≥20 years, p=0·050.

**Figure 4:**
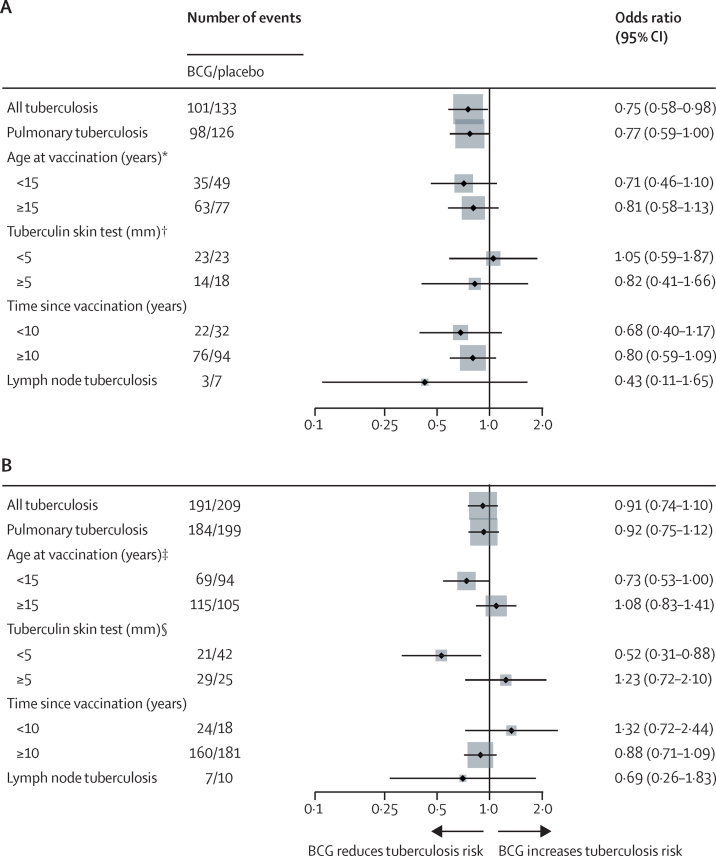
BCG *vs* placebo in scar-positive individuals, by HIV status HIV negative (A). HIV positive (B). Intention-to-treat population for certain and probable tuberculosis. *p_interaction_=0·65. † p_interaction_=0·60. ‡p_interaction_=0·059. §p_interaction_=0·024.

Additional exploratory analyses examined the relationship between age at vaccination, years since vaccination, and effects on HIV-negative tuberculosis. The protective effect of repeat BCG on pulmonary tuberculosis was only seen in those vaccinated at younger than 15 years, and followed up for at least 20 years (figure 3). Separating the analyses by HIV status (figure 4) reveals lower ORs (further from 1) among HIV-negative individuals for all subgroups, with the exception of those with tuberculin reactivity of less than 5 mm, for whom the OR was lower in HIV-positive individuals (0·52 [0·31–0·88]).

For the per-protocol analysis, 3888 individuals (60 of whom developed tuberculosis) were excluded, mostly due to doubtful scar status (details in appendix p 9). The OR for all tuberculosis was 0·87 (0·76–1·01) and 0·88 (0·76–1·02) for pulmonary tuberculosis (appendix p 14). The results by TST status, age, years since vaccination, HIV status, and lineage were similar to the main analysis.

When the outcome was changed to diagnostically certain tuberculosis, the ORs were 0·95 (0·80–1·13) for all tuberculosis (538 cases) and 0·98 (0·82–1·17) for pulmonary tuberculosis (503 cases) in the intention-to-treat analysis, as shown in the appendix (p 8). In the per-protocol analysis of certain tuberculosis, the ORs were 0·90 (0·76–1·07) for all tuberculosis (504 cases) and 0·93 (0·77–1·11) for pulmonary tuberculosis (470 cases), as shown in the appendix (p 11).

**Figure 5:**
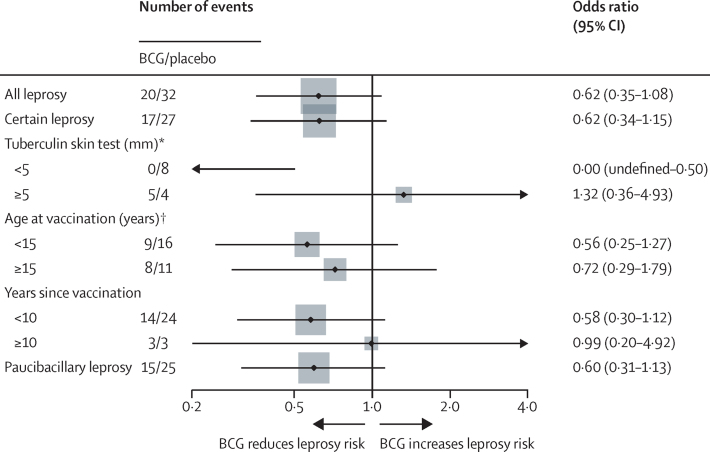
Odds ratios of leprosy associated with repeated BCG among scar-positive individuals allocated either repeat BCG or placebo (intention-to-treat population) Note, for subgroup <5 mm one-sided 95% CI calculated (Cornfield method). *p value for interaction=0·013. † p value for interaction=0·95.

Only 44 diagnostically certain leprosy cases arose among the scar-positive individuals who received either BCG or placebo, 35 of whom were diagnosed by 1995 and only nine in the years since. Figure 5 shows results for leprosy, indicating approximately 40% protection overall over 30 years, but none after 10 years post-vaccination. There is an indication of strong protection in those who were tuberculin-negative at time of repeat vaccination (0 leprosy events in the BCG group versus eight in the placebo group; p=0·0079).

The appendix describes the results of the analyses of BCG plus killed *M leprae* vaccines versus both tuberculosis and leprosy.

## Discussion

In this long-term follow-up of the double-blind, randomised, placebo-controlled trial of repeat BCG in Karonga District, Malawi, we found no evidence of protection against pulmonary tuberculosis overall, and the CIs for lymph node tuberculosis are very wide. There are indications of protection in some subgroups. Although these analyses were predefined, the results need to be interpreted cautiously given the large number of comparisons that were made.

We hypothesised that there might be more protection in those vaccinated as children because they would have been less exposed to tuberculosis and to the mycobacterial infections that are thought to mask the benefit of BCG.^9^, ^25^ In the 1995 analysis, most of these individuals were still in age groups in which tuberculosis disease is rare, as there are very few cases of childhood tuberculosis diagnosed and confirmed in this population (appendix p 6). As predicted, there was some evidence of protection in those who were children at repeat vaccination that was not evident in the earlier analysis. This was only apparent in those with the longest follow-up, providing an explanation for the protection by repeat BCG in this group.

The delayed effect after childhood vaccination is in line with the results of the BCG revaccination trial in Brazil, in which evidence of protection was only seen after longer follow-up and in the youngest children.^5^, ^6^ A similar pattern was seen in the large south India trial in Chingleput, comparing BCG and placebo in a BCG-naive population, in which the initial analysis, done after 7·5 years, found no evidence of protection, whereas low but consistent protection against tuberculosis was recognised in those vaccinated at less than 15 years of age after 15 years of follow-up.^10^, ^11^

The tuberculosis ascertained in the trial over 30 years was overwhelmingly in adults, and 63% of those tested were HIV positive (appendix p 6). There was some evidence for modest protection by repeat BCG against HIV-negative tuberculosis (OR=0·77; 0·59–1·00). Although it would be highly valuable to have a vaccine which could protect against HIV-positive tuberculosis disease, no currently available product can achieve this, and all efficacy estimates to date for BCG vaccines have been against HIV-negative tuberculosis. There was no evidence of an increase in risk of HIV-positive tuberculosis with repeat BCG (as had been suggested on the basis of the small numbers in the 1995 analysis).^8^

The fact that the Karonga trial did not exclude tuberculin-positive participants has been suggested as a possible explanation for the lack of protection by BCG found in the earlier analysis.^2^ There is no evidence that BCG can protect if administered after *M tuberculosis* infection (many trials have excluded tuberculin reactors) and much evidence that protection can be masked by previous exposure to environmental mycobacteria.^9^, ^25^ TST reactivity was only known for 37% of the participants, limiting the power of this analysis. There was little evidence of protection in those with reactions of less than 5 mm (OR=0·83, with a wide CI 0·59–1·16). As all of these participants had received BCG previously, those without tuberculin reactivity at the time of repeat vaccination had either failed to convert after their initial vaccination (were these perhaps primary vaccine failures?) or else had reverted at some time after the initial vaccination. We have noted elsewhere that such reversion is common in the Karonga population.^18^

Among other explanations that have been given for differences in BCG's effectiveness between populations has been the suggestion that it might be attributable to differences in circulating strains of *M tuberculosis*.^12^, ^13^ We found no evidence of differential protection by genotype, but the numbers for lineage 2 (Beijing genotype) were very small.

There were only 37 cases of lymph node tuberculosis ascertained over the 30 years. Although the low OR (0·6) had a wide associated 95% CI (0·31–1·17), we note that BCG has generally been found to be more effective against extrapulmonary than against pulmonary tuberculosis.^9^, ^26^

We used bacteriologically or histologically confirmed tuberculosis as the endpoint to minimise misclassification. 65% of cases met the even stricter definition of certain tuberculosis. Restricting to this group gave similar results. Our main analysis was intention to treat. The per-protocol analysis, which excluded those with doubtful BCG scars as well as the few with discrepant vaccine scar reports and vaccine codes, also gave similar results, although with ORs slightly further from 1 (appendix p 8).

The 49% protection against leprosy reported for repeat BCG in 1996 was similar to the protection imparted against leprosy by a single dose of BCG, as measured in case-control and cohort analyses in this population, before the trial.^27^, ^28^ Only nine new cases were ascertained over the 23 years since the initial analyses, among those randomly assigned to repeat BCG or placebo, and there was no evidence of any protection beyond 10 years post-vaccination (OR=0·99). The significant evidence of protection among those who were tuberculin-negative at time of repeat vaccination is consistent with several observations of BCG's effectiveness being highest in those tuberculin negative at time of vaccination. (It is for this reason that many trials have excluded tuberculin positives.)^9^ The decline in leprosy numbers is itself of interest. It might reflect in part a decline in ascertainment sensitivity, but also reflects the progressive fall in leprosy incidence observed in many countries in recent decades).^29^ In the Karonga context this decline has been evident since 1980. It is likely to have been influenced by the control programme introduced into the population by the British Leprosy Relief Association (Lepra) in 1974, and aggressive case finding and treatment within the context of two total population surveys by the Lepra Evaluation Project in the 1980s.^17^, ^30^ Beyond that, the decline was also encouraged by the introduction of BCG within the Expanded Programme on Immunisation in the late 1970s and the fact that the Karonga Prevention Trial ensured that almost everyone had received at least one dose of BCG, and many had received two doses. The Karonga population might in fact have received the most intensive leprosy control efforts of any leprosy endemic population in the world, with the evident result that the incidence has been reduced to near zero.

The Karonga trial recruited without restriction by age or previous TST status because an important motive was to evaluate protection by repeating a BCG vaccination against disease in all ages; and it was planned and initiated before HIV was known in the population. The identification procedures established more than 40 years ago have allowed individuals with diagnosed tuberculosis and leprosy in the district to continue to be linked reliably to the trial population.^30^ All project staff remained masked to the interventions throughout the follow-up. Passive follow-up means that the incidence of tuberculosis and leprosy is doubtless underestimated. But as the vaccine groups were randomly assigned, this should not introduce bias into the relative risks reported here. The data from the demographic surveillance area within the district confirm that there was no differential mortality or emigration from the area by vaccine group (appendix p 3). We thus present these analyses in considerable detail, here and in the appendix, as this long and detailed follow-up provides a unique resource for the continued effort to develop and evaluate mycobacterial vaccines.

In conclusion, we find no evidence of a strong protective effect of repeat BCG on all tuberculosis in this rural African population, with a high prevalence of HIV, but a suggestion of modest protection against HIV-negative tuberculosis, and of protection when the second vaccine was given in childhood. Protection against leprosy was strongest in individuals without tuberculin reactivity at time of vaccination, but appeared to decline by 10 years post-vaccination. These patterns, based on the longest detailed follow-up of a mycobacteria vaccine trial to date, are consistent with much that is known about BCG and cumulative exposure to mycobacterial antigens, from trials and observational studies, and should be considered in the planning, analysis, and interpretation of future mycobacterial vaccine trials.

## Data sharing

On publication, de-identified individual participant data that underlie the results reported in the Article will be made available via Datacompass. Proposals should be directed to Chifundo.Kanjala@LSHTM.ac.uk; to gain access, data requestors will need to sign a data access agreement.

## Declaration of interests

JRG, KF, and PEMF received grant funding from the Gates Foundation for this work. All other authors declare no competing interests.
