# Supplementary material for: BCG re-vaccination in Malawi: 30-year follow-up of a large, randomised, double-blind, placebo-controlled trial
Source: Lancet Glob Health. 2021 Sep 14;9(10):e1451–9. doi: 10.1016/S2214-109X(21)00309-0 (PMC8459381; doi:10.1016/S2214-109X(21)00309-0)
Supplement: Supplementary appendix [file mmc1.pdf]

THE LANCET
Global Health

Supplementary appendix

This appendix formed part of the original submission and has been peer reviewed. We post it as supplied by the authors.

Supplement to: Glynn JR, Fielding K, Mzembe T, et al. BCG re-vaccination in Malawi: 30-year follow-up of a large, randomised, double-blind, placebo-controlled trial. *Lancet Glob Health* 2021; **9:** e1451–59.

Supplementary appendix

30-year follow-up of a large randomised double-blind placebo-controlled trial of BCG revaccination in Malawi

Contents

1. Further notes on background to the trial
2. Surveillance and follow-up by vaccine group
3. Further details on tuberculosis data and results
   - 3.1 Case definitions
     - 3.1.1 Diagnostic certainty criteria for tuberculosis
     - 3.1.2. Criterion for postvaccination status of tuberculosis cases
   - 3.2 CONSORT flow chart
   - 3.3 Repeat BCG vs placebo among scar positives
     - 3.3.1 Intention to treat
     - 3.3.2 Per protocol
   - 3.4 Evaluation of BCG plus killed *M leprae* vaccines
     - 3;4.1 In scar negatives
     - 3.4.2 In scar positives
     - 3.4.3 In scar negative and scar positive individuals combined
     - 3.4.4 Combining BCG and BCG plus killed M leprae versus placebo, in scar positives
     - 3.4.5 Discussion
4. Further details of leprosy data and results
   - 4.1 Case definitions
   - 4.2 CONSORT flow chart
   - 4.3 Repeat BCG vs placebo among BCG scar-positives
   - 4.4 Evaluation of BCG plus killed *M leprae* vaccines
   - 4.5 Discussion
5. References
6. Karonga Prevention Trial Group

1. Further notes on background to the trial

The Karonga Prevention Trial (KPT) recruitment took place 1986-1989, having been planned in the early 1980s. The proposal was driven initially by leprosy interests, led by the Immunology of Leprosy (IMMLEP) Steering Committee of the WHO TDR Programme. Thus the trial was based within a large leprosy project, known first as the Lepra Evaluation Project and later as the Karonga Prevention Study, which had been evaluating serological and skin tests developed through the IMMLEP programme since 1979.[1-4] This project had incorporated tuberculosis case finding, and its laboratory provided sputum smear services since 1980, introducing culture in 1986.[5] There was thus a framework for a trial with tuberculosis as well as leprosy outcomes. Several studies had already shown that BCG vaccines could impart a variable degree of protection against leprosy, in different populations, and there was particular interest in the IMMLEP programme as to whether the addition of leprosy bacillus antigens might enhance this protection .[6] The project also provided an opportunity to evaluate giving a second BCG vaccination, a policy which was then practiced for tuberculosis control in several countries, but which had never been evaluated in a randomised controlled trial. The trial was designed and initiated before HIV was recognised in the trial population.

BCG vaccination had been introduced into Karonga District in the late 1970s, first in campaigns which targeted schoolchildren, and then as part of the routine childhood immunization programme organised by the EPI .[7] There was evidence from case control and cohort analyses that a single BCG vaccine imparted approximately 50 % protection against leprosy, but no protection against tuberculosis in the Karonga population .[5,8]

The original design was for four groups: BCG versus BCG plus killed *M leprae* vaccines in individuals lacking scar evidence of prior BCG vaccination, and BCG versus placebo in individuals with scar evidence of prior BCG. Recruitment began in January 1986 with this basic design, with the combined vaccine including 5 x 10^7 killed *M leprae* per dose. This was changed, on recommendation of the WHO IMMLEP programme, in July 1986, when a higher dose of killed *M leprae* (6 x 10^8 bacilli) was introduced, first for the scar negative participants, and later (in August 1987) as an additional arm among scar positive individuals. This change was introduced after pilot investigations had shown that the higher dose was safe .[6] The *M leprae* suspension vaccine was prepared at the Wellcome Laboratories, Beckenham, UK.[9,10]

2. Surveillance and follow-up by vaccine group

Follow-up of the trial population was through a mixture of active and passive surveillance. The active surveillance component consisted of annual house-to-house surveys, carried out 1991 – 1994, in four areas of the district each with 5,000 – 10,000 population, known to have had a relatively high incidence of leprosy. In addition, all individuals who were either first degree

relatives of, or known to have lived in the same house as, a known leprosy patient since 1980, were followed up in early 1995, wherever they then lived, and examined. Furthermore, 19,471 participants in the trial lived in the area selected for a demographic surveillance site (the Karonga Continuous Registration System), established in 2002.[11] The initial survey for this surveillance site included an effort to trace all individuals who were known to have lived in the area at the time of trial recruitment 1986-1989, and recorded dates of death and emigration. **Figure S-1** presents the survival and emigration rates of each of the vaccine groups in the surveillance area since date of vaccination, through 2018, showing no difference between the vaccine groups.

Figure S-1: Proportions who died (A, B) or emigrated (C, D) by vaccine group, by time since vaccination, among trial participants resident in the Karonga demographic surveillance site area.

A

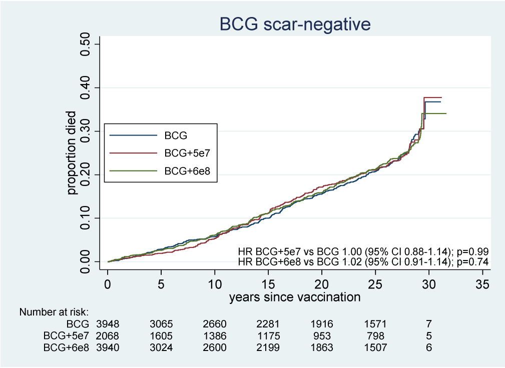

B

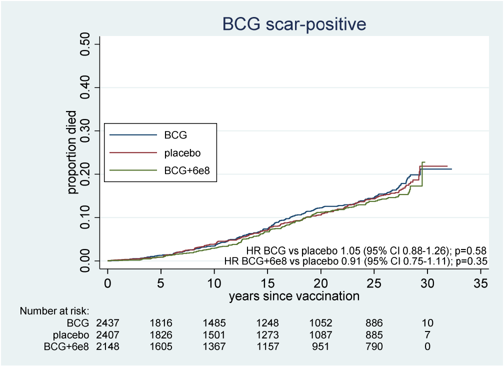

C

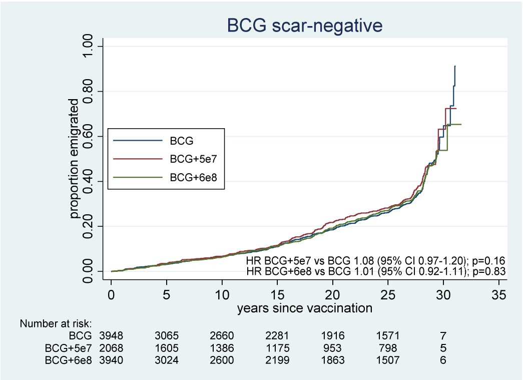

D

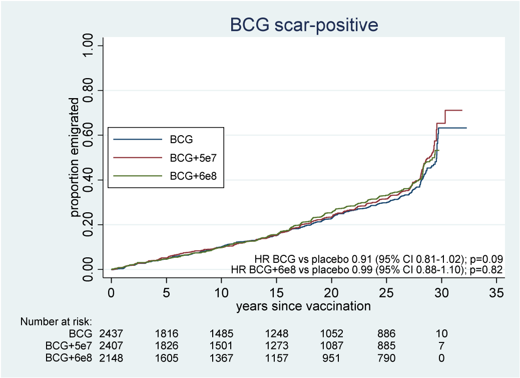

(Note: the up-turns at the ends of each graph are an artefact due to events (deaths or migrations) being reported and recorded in the dataset before each re-census)

3. Further details on tuberculosis data and results

3.1 Tuberculosis case definitions

Changes in laboratory methods have required a change in tuberculosis case definitions compared to those used in the 1995/6 analysis. The following changes were agreed by the Data Monitoring Committee for the 2020 analyses.

3.1.1 Diagnostic certainty criteria for tuberculosis

In 1995/6, the following definitions were used:

> "The diagnosis of pulmonary tuberculosis was counted as certain when an individual had a positive sputum culture and at least one other sputum specimen positive on either culture or microscopy. Probable cases were all other patients with positive sputum bacteriology results: except those with only one positive smear with fewer than 10 bacilli per 100 fields, who were excluded. The diagnosis of extrapulmonary tuberculosis was based on clinical signs, biopsy samples, and bacteriological results; biopsy or culture evidence was required for a case to be counted as certain". [10]

Because of the introduction of GeneXpert technology in the district hospital in 2013, the fact that we have culture confirmation only until 31 December 2015, and the addition of data from genotyping, these definitions have been revised as follows:

> *Pulmonary*: certain (C) if culture positive or GeneXpert positive or genotyping (spoligotype or restriction fragment length polymorphism or whole genome sequence) shows *M tuberculosis*, plus at least one other specimen positive on culture or GeneXpert or genotyping or microscopy; probable (P) if culture or Xpert or genotyping or microscopy positive but not fulfilling criteria for certain, and excluding those with only a single scanty smear (ie fewer than 10 bacilli per 100 fields). (Note that smears for which the corresponding culture or genotype shows non-tuberculous mycobacteria are not included, and the only patient with *M bovis* was excluded.)
>
> *Extrapulmonary*: certain (C) if histology "certain" or aspirate is positive on microscopy or culture/Xpert/genotype (excluding single scanty smear); probable (P) if histology "probable" or clinical lymph node tuberculosis and the aspirate has a scanty positive smear.
>
> **Note:** Pulmonary means pulmonary +/- other sites
>
> "Lymph node" means involving lymph nodes with or without other extrapulmonary sites, but without pulmonary involvement.

3.1.2 Criterion for postvaccination status of tuberculosis cases

In 1995/6, the following definition was used:

> "A case was considered as postvaccination if there was no recorded evidence of tuberculosis until at least 60 days after vaccination.".[10]

In that analysis there was an interest in the possible precipitation of TB by BCG vaccination, so any early cases (between 60 days and 6 months) were important to include if we had seen them previously without evidence of TB. This required individual checks of such cases. No such increase in early cases was found associated with vaccine, so there is no need to explore this again. By removing the need to have seen people prior to vaccination we can include some cases who were previously excluded. And a 6-month cut-off for all gives more certainty that the cases are actually post-vaccination. The following definitions are thus used in the current analyses:

- All cases occurring within 6 months (182 days) of vaccination are excluded.
- All cases with a history of TB prior to vaccination (or at an unknown date) by either documentation or self-report are excluded.
- Only the first episodes of certain or probable TB after vaccination are included

3.2 CONSORT flow chart

The CONSORT flow chart for the complete trial with reference to tuberculosis outcomes, for both the intention to treat (ITT) and per protocol (PP) analyses, is presented in the main paper as Figure 1.

3.3 Repeat BCG and placebo comparison

The distributions of pulmonary tuberculosis cases by age and HIV status at time of diagnosis are shown for the placebo and BCG recipients in **Figure S-2**

Figure S-2: Distributions of pulmonary tuberculosis cases by age and HIV status at time of TB diagnosis.

a. placebo (404)

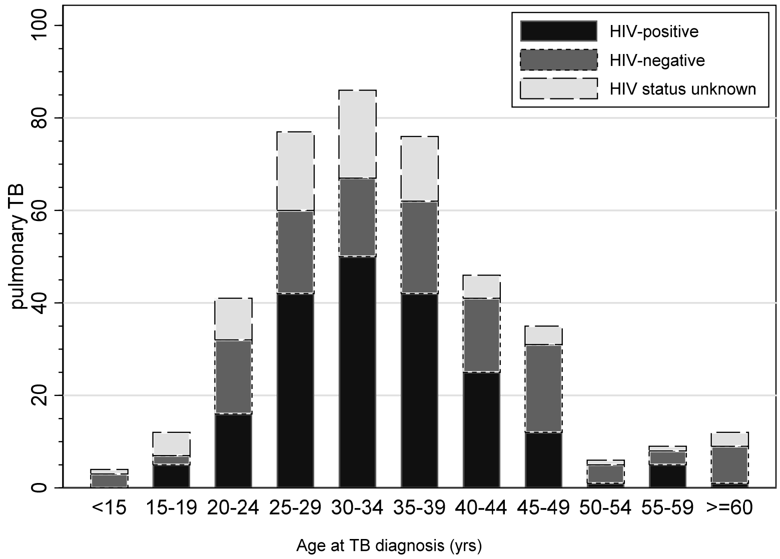

b. BCG (382)

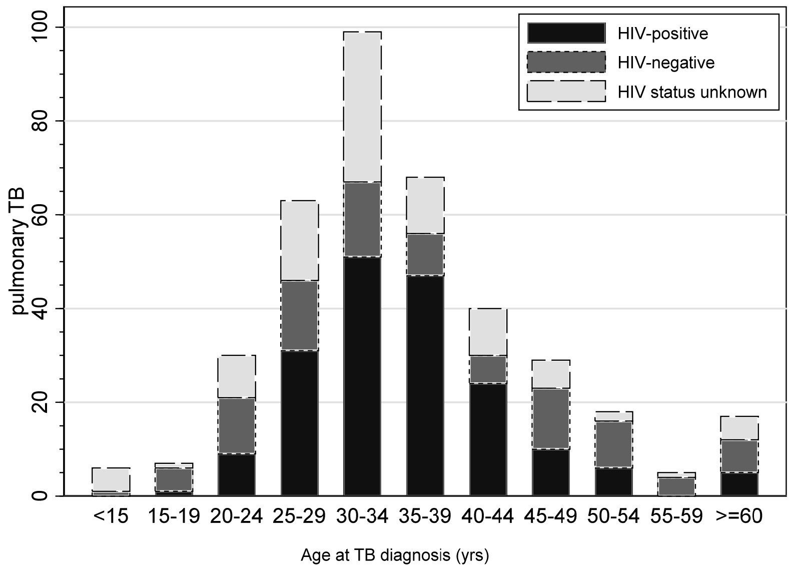

3.3.1 Intention to treat analysis

The flow chart for the intention to treat analysis is in the main paper (Figure 1), along with the odds ratios for the various comparisons, for all 824 certain and probable tuberculosis cases (Figure 2). The distributions by study arm for age at vaccination, sex and TST status at vaccination are shown below, in **Table S-1**. The odds ratios for the 538 *diagnostically certain* tuberculosis cases, among scar positive individuals allocated either repeat BCG or placebo, are shown in **Figure S-3**.

Table S-1: Baseline characteristics at time of vaccination, for tuberculosis analysis of placebo versus BCG, by study arm (intention-to-treat population)

Factor	Level	placebo	BCG
**N - individuals**		**23,330**	**23,502**
Age, grouped (years)	≤4	3541 (15.2%)	3597 (15.3%)
	5-14	9697 (41.6%)	9687 (41.2%)
	15-24	5916 (25.4%)	5902 (25.1%)
	≥25	4176 (17.9%)	4316 (18.4%)
Age, median (IQR) (years),		13.0 (7.0, 22.0)	13.0 (7.0, 22.0)
Sex, female		11802 (50.6%)	11849 (50.4%)
TST, grouped (mm)	<5	5460 (62.3%)	5201 (62.5%)
	5-16.9	2796 (31.9%)	2609 (31.3%)
	≥17	511 (5.8%)	517 (6.2%)
Unknown TST		14563 (62.4%)	15175 (64.6%)

IQR interquartile range; TST tuberculin skin test

Figure S-3: Odds ratios of *diagnostically certain* tuberculosis associated with repeated BCG, among scar positive individuals allocated either repeat BCG or placebo (ITT).

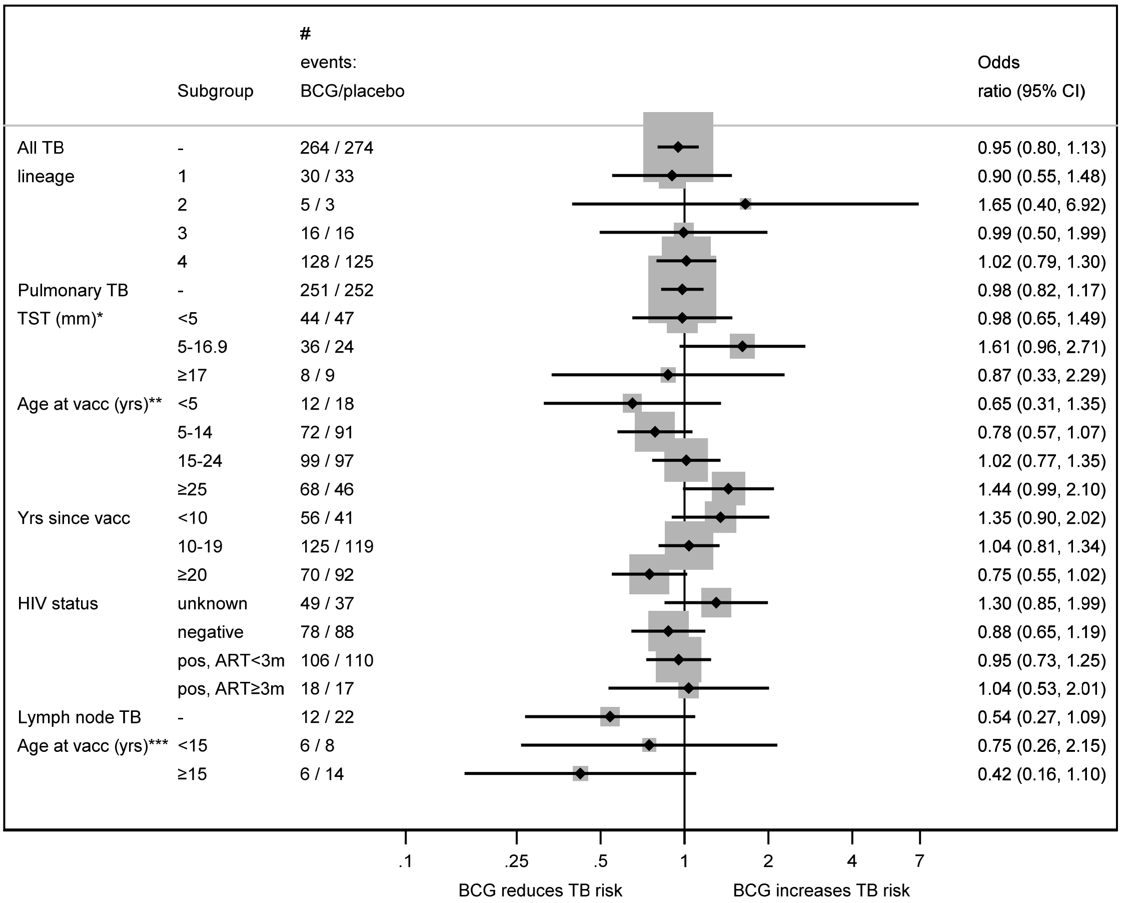

*interaction P-value 0.3; **interaction P-value 0.06; ***interaction P-value 0.4

3.3.2 Per protocol analysis

Table S-2 presents the study population numbers for the per protocol analysis of BCG versus placebo in scar positives. Odds ratios are shown for certain and probable tuberculosis in **Figure S-4**. Compared with the ITT analysis (Figure 2, main paper), the odds ratios are very similar. Interestingly, the result in HIV negatives is slightly stronger (0.70; 0.57 – 0.93) than in the ITT analysis (0.77; 0.59 – 1.00). Odds ratios for diagnostically certain tuberculosis in the per protocol analysis are presented in **Figure S-5**, being 0.8 (0.58-1.10) in HIV negatives.

Table S-2: Study population for per protocol analysis of BCG versus placebo in scar positives

Factor	Level	placebo	BCG
**N**		**21378**	**21566**
Age, grouped (years)	<5	3323 (15.5%)	3377 (15.7%)
	5-14	8873 (41.5%)	8882 (41.2%)
	15-24	5674 (26.5%)	5627 (26.1%)
	≥25	3508 (16.4%)	3680 (17.1%)
Age (years), median (IQR)		12.0 (6.0, 21.0)	12.0 (7.0, 22.0)
Sex, female		10716 (50.1%)	10821 (50.2%)
TST, grouped (mm)	<5mm	5020 (62.2%)	4777 (62.2%)
	5-16.9mm	2587 (32.1%)	2435 (31.7%)
	≥17mm	458 (5.7%)	470 (6.1%)
Missing TST		13313 (62.3%)	13884 (64.4%)

Figure S-4: Odds ratios for certain and probable tuberculosis associated with repeated BCG, among scar positive individuals allocated either repeat BCG or placebo (per protocol analysis).

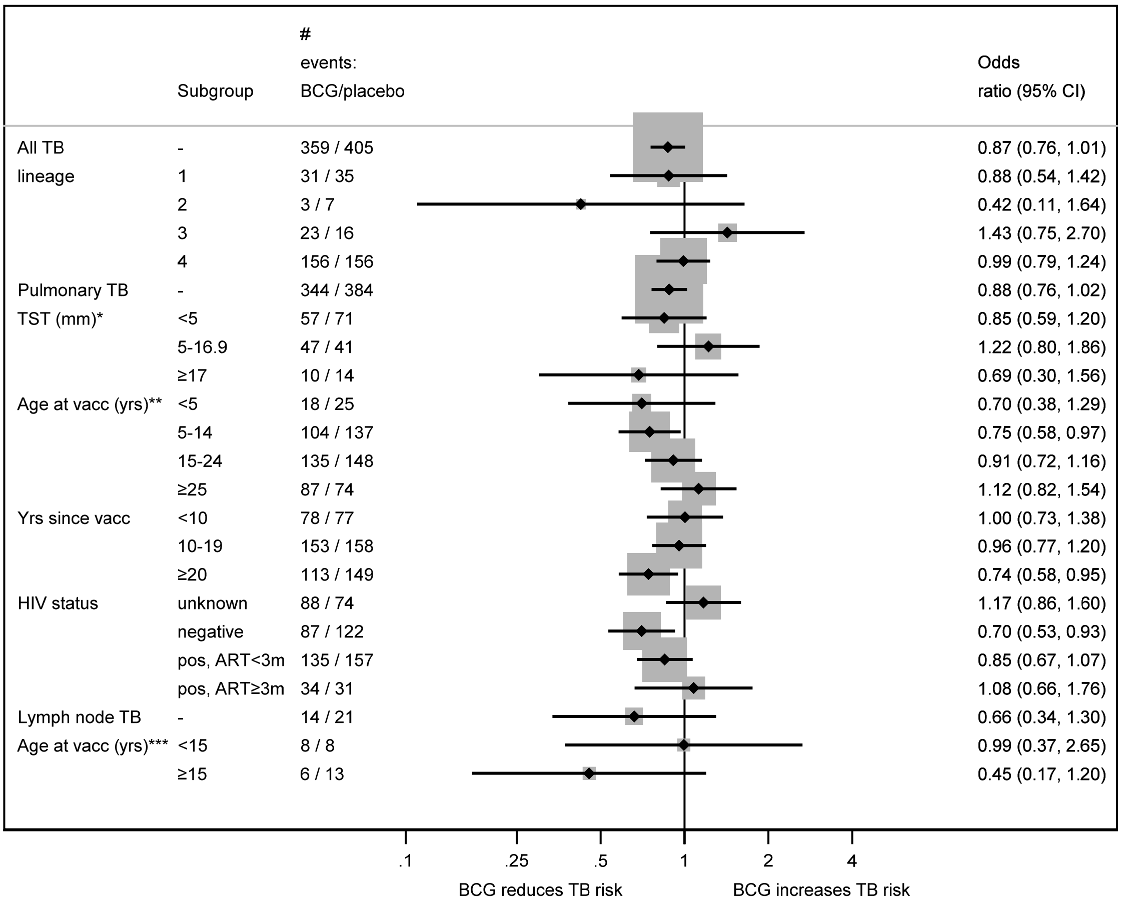

*interaction P-value 0.3; **interaction P-value 0.2; ***interaction P-value 0.3

Figure S-5: Odds ratios for *diagnostically certain* tuberculosis associated with repeated BCG, among scar positive individuals allocated either repeat BCG or placebo (per protocol analysis).

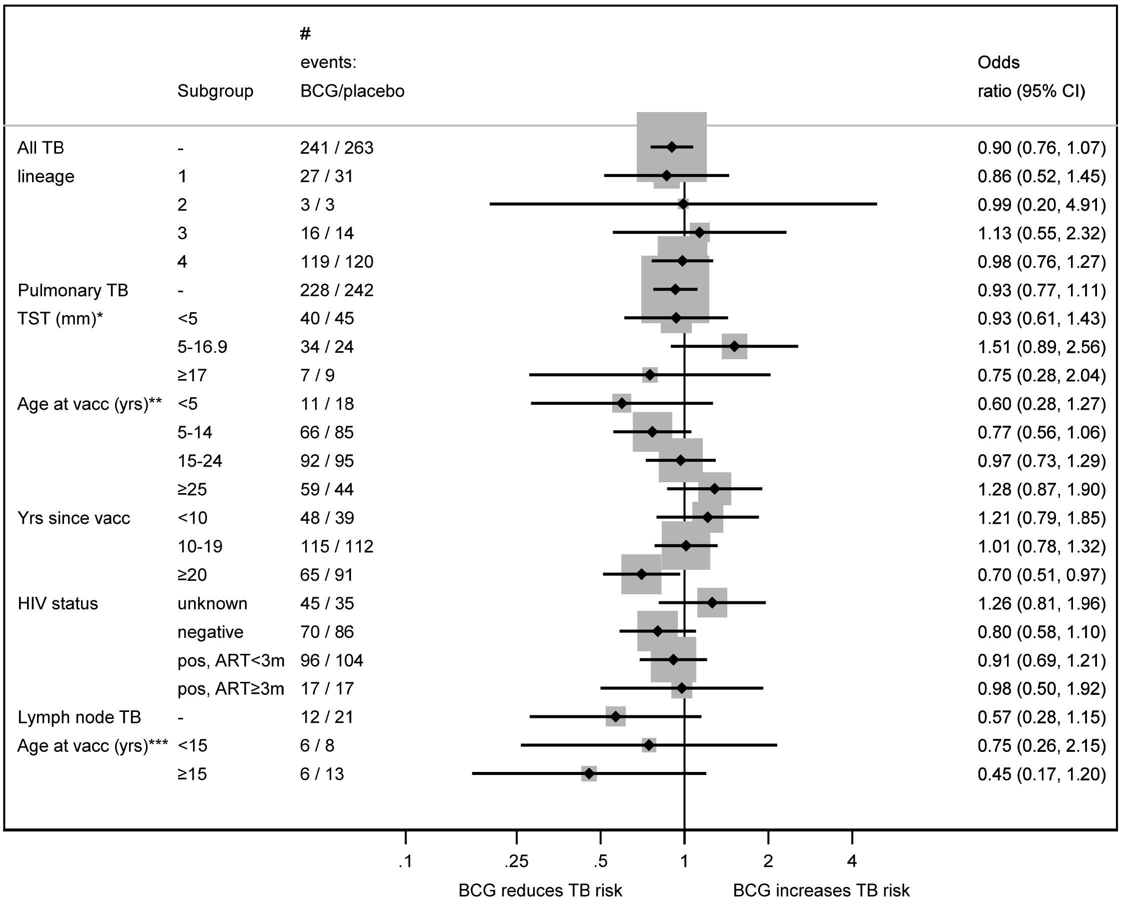

*interaction P-value 0.3; **interaction P-value 0.1; ***interaction P-value 0.5

3.4 Evaluation of BCG plus killed *M leprae* vaccines against tuberculosis

The BCG plus killed *M leprae* vaccines were developed and incorporated into the trial with intention to prevent leprosy, but it is appropriate to examine the results of these vaccines also versus tuberculosis, given that they represent the addition of mycobacterial antigens to BCG, and there is evidence of heterologous protection against tuberculosis associated with environmental mycobacteria.[12]

The evaluation of the combined BCG plus killed *M leprae* vaccine was made complicated by the change in design described in Section 1, above. Not only were there two doses of killed *M leprae* (5×10^7 and 6×10^8) but a combined vaccine arm was introduced for scar positives. Three comparisons were thus agreed for the data analysis plan by the Data Monitoring Committee:

- between BCG plus killed *M leprae* versus BCG alone in scar negative individuals;
- between BCG plus killed *M leprae* versus BCG alone in scar positive individuals; and
- between BCG plus killed *M leprae* versus BCG alone in both scar negative and scar positive individuals combined.

The analysis plan also proposed (as in 1995), that, if no difference was observed between the BCG and BCG plus killed *M leprae* vaccines, then it would be appropriate to combine the BCG and BCG plus killed *M leprae* groups and compare them against the placebo group, all in scar positives.

In order to ensure these comparisons are made between groups vaccinated concurrently, the analyses were stratified by time period when the compared vaccines were used.

3.4.1 In scar negative individuals

Table S-3 presents the ITT study population for the comparison of BCG versus BCG plus killed *M leprae* among individuals with no BCG scar at time of vaccination. Odds ratios are shown in **Figure S-6**. Adjustment for time period before versus after change of dose of killed *M leprae* (01 July 1986) made very little difference: Odds ratios for all TB, pulmonary TB and lymph node TB were 0.95 (0.84 – 1.08), 0.94 (0.83 – 1.07) and 1.45 (0.70 – 3.01) respectively.

Table S-3 Study population for analysis of BCG plus killed *M leprae* vs BCG, among BCG scar-negatives (ITT)

Factor	Level	BCG	BCG+KML
N		27851	38162
Age, grouped (years)	<5	5851 (21.0%)	8278 (21.7%)
	5-14	6694 (24.0%)	8953 (23.5%)
	15-24	3124 (11.2%)	4285 (11.2%)
	≥25	12182 (43.7%)	16646 (43.6%)
Age (years), median (IQR)		17.0 (6.0, 41.0)	17.0 (6.0, 42.0)
Sex, female		14918 (53.6%)	20426 (53.5%)
TST, grouped (mm)	<5	6310 (64.0%)	8808 (64.0%)
	5-16.9	2759 (28.0%)	3931 (28.6%)
	≥17	792 (8.0%)	1024 (7.4%)
Missing TST		17990 (64.6%)	24399 (63.9%)

Figure S-6: Odds ratios for certain and probable tuberculosis comparing BCG plus killed *M leprae* versus BCG vaccine among scar negative individuals (ITT).

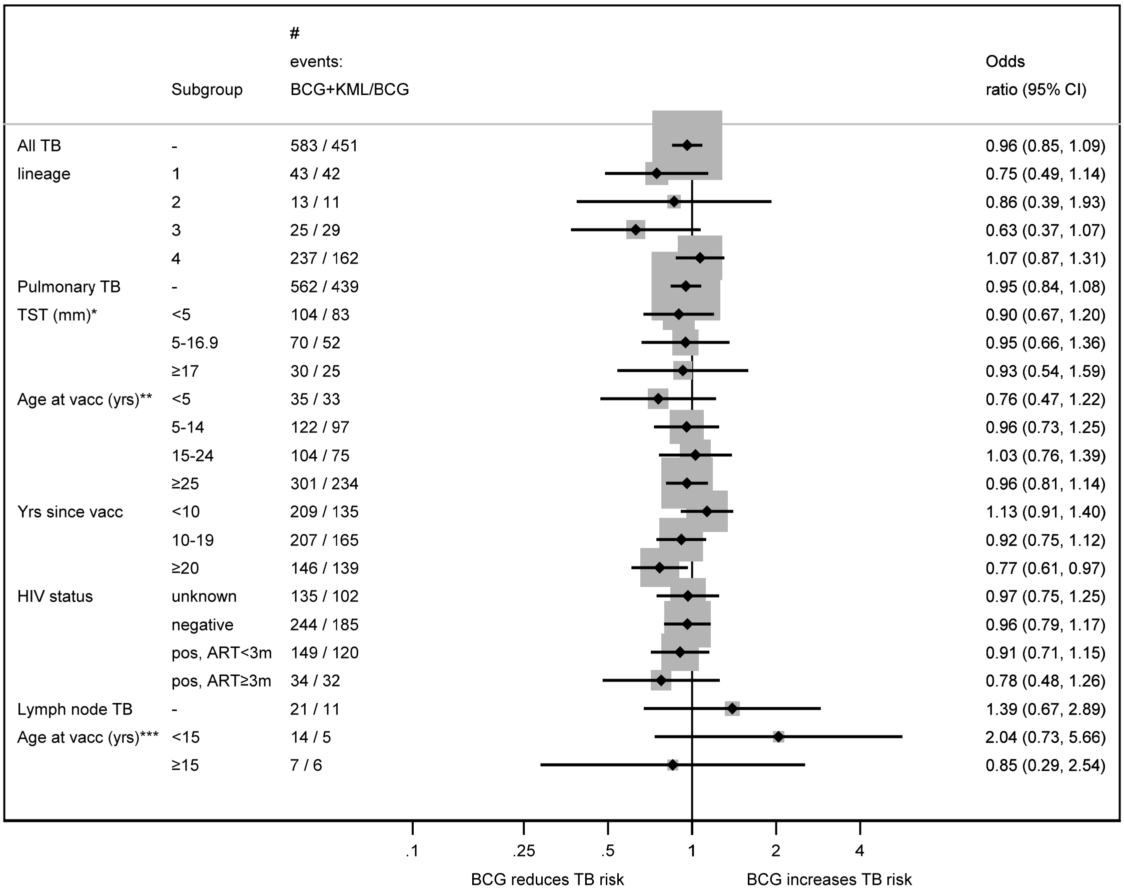

*interaction P-value >0.9; **interaction P-value 0.8; ***interaction P-value 0.2

3.4.2 In scar positive individuals

Table S-4 presents the ITT study population for the comparison of BCG versus BCG plus killed *M leprae* among individuals with a BCG scar at time of vaccination. Odds ratios are shown in **Figure S-7.**

Table S-4 Study population for analysis of BCG+KML vs BCG, among BCG scar-positives (ITT)

Factor	Level	BCG	BCG+KML
N		23502	8140
Age, grouped (years)	< 5	3597 (15.3%)	1082 (13.3%)
	5-14	9687 (41.2%)	3425 (42.1%)
	15-24	5902 (25.1%)	2150 (26.4%)
	≥25	4316 (18.4%)	1483 (18.2%)
Age (years), median (IQR)		13.0 (7.0, 22.0)	13.0 (7.0, 22.0)
Sex, female		11849 (50.4%)	4107 (50.5%)
TST, grouped (mm)	<5	5201 (62.5%)	1786 (59.6%)
	5-16.9	2609 (31.3%)	1146 (38.2%)
	≥17	517 (6.2%)	67 (2.2%)
Missing TST		15175 (64.6%)	5141 (63.2%)

Figure S-7: Odds ratios for certain and probable tuberculosis comparing BCG plus killed *M leprae* versus BCG vaccine among scar positive individuals (ITT).

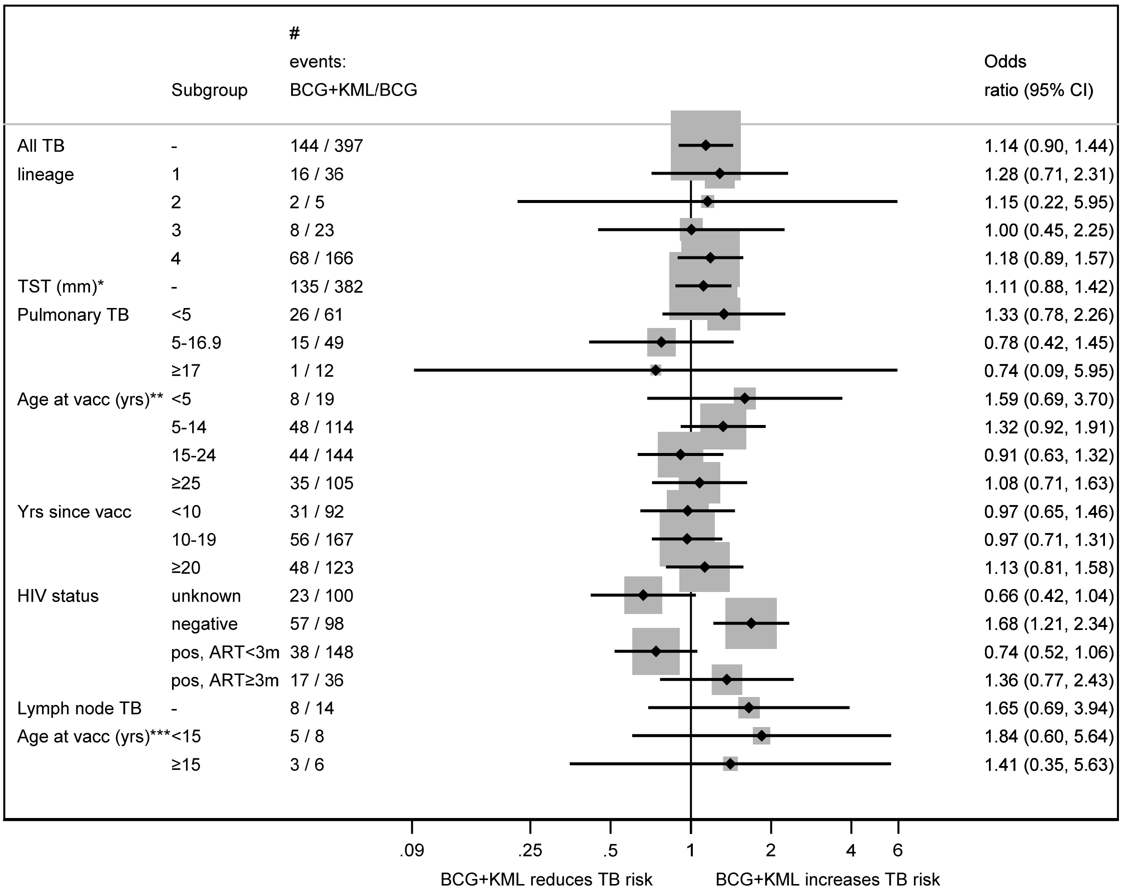

*interaction P-value 0.3; **interaction P-value 0.4; ***interaction P-value 0.8

3.4.3 In scar negative and scar positive individuals combined

The odds ratio for pulmonary tuberculosis, comparing BCG plus killed *M leprae* to BCG, combining all data in scar negatives and scar positives is 0.95 (0.86 – 1.06), indicating no overall effect (**Table S-5**).

Table S-5 Odds ratios for certain and probable tuberculosis combining comparisons between BCG plus killed *M leprae* versus BCG in both scar negatives and scar positives, (ITT).

Vaccine	N	# PTB	row %	OR	lower 95%	upper 95%
BCG+KML	46,302	697	1.51%	0.95	0.86	1.06
BCG	51,353	821	1.60%	1		

3.4.4 Combining BCG and BCG plus killed *M leprae* versus placebo, in scar positives

Given no evidence that the addition of killed *M leprae* antigens influences the protection imparted by BCG alone against tuberculosis (**Table S-5**), we combine the BCG and BCG+ killed *M leprae* groups and compare them to placebo recipients in scar positives (**Table S-6**). There is no evidence of protection overall against tuberculosis overall in this combined analysis: OR= 0.95 (0.83 – 1.09). This was the same after adjustment for time period before and after 01 July 1986.

Table S-6: The odds ratio for pulmonary tuberculosis, combining BCG and BCG plus killed M leprae together, against placebo, in scar positives:

Vaccine	N	# PTB	row %	OR	lower 95%	upper 95%
BCG+KML / BCG	31,642	517	1.63%	0.95	0.83	1.09
placebo	23,330	404	1.73%	1		

3.4.5 Discussion

We again encourage caution in interpretation, given the multiple comparisons involved in these analyses. Regarding the implications of adding killed *M leprae* antigens to BCG, the overall impression is of no consistent or significant effects in these analyses, as evident in 3.4.3 which included both scar negatives and scar positives. Trends by age and by time since vaccination appear to go in the opposite direction in the scar positive and scar negative groups, and an apparent increased risk of HIV negative tuberculosis associated with BCG+KML compared to BCG alone in those with BCG scars (**Figure S-7**) is not seen in those without scars (**Figure S-6**). We conclude that the addition of *M leprae* antigens to BCG vaccine had no observable effect on protection against tuberculosis. To the best of our knowledge this trial is the only published examination of the efficacy of BCG plus killed *M leprae* vaccines against tuberculosis.

4. Further details of leprosy data and results

4.1 Ascertainment and diagnosis of leprosy

The trial took place within a large leprosy project which had begun in 1979.[13] Much effort had been devoted to establishing rigorous diagnostic criteria for leprosy, leading to an algorithm for diagnostic certainty based upon clinical findings, a clinical certainty score given by a medical officer, and an independent certainty assessment by a histopathologist.[13] This algorithm was used for diagnostic confirmation for all the leprosy cases in the trial analyses.

The first trial analysis took place in late 1995, after 6 – 9 years of follow up, at which time 107 post-vaccination leprosy cases had been ascertained in the entire trial population. Of these, 93 were considered diagnostically certain.[10] Compared to placebo among participants with a BCG scar, the second BCG imparted 49 % protection against diagnostically certain paucibacillary and multibacillary leprosy (35 cases, RR=0.51; 95 % CI=0.25 -1.03). Given that there was observational study evidence that a single BCG imparted at least 50 % protection against leprosy in this population, this implied that two BCG vaccinations could induce some 75% protection. The fact that both the case-control and cohort observational studies had shown that protection was stronger against diagnostically certain than against certain and probable cases combined provided the rationale to focus these analyses on diagnostically certain cases. This was stipulated explicitly in the plans for the 1995 and most recent analyses. Analysis by age in 1995 had suggested greater protection among those under 15 years of age at vaccination (21 cases; RR=0.4; 95%CI=0.15 – 1.01). There was no analysis in 1995 by tuberculin status at time of vaccination.

Ascertainment of leprosy continued to be based upon passive surveillance after 1995, including biopsy of all individuals suspected to have leprosy until 2000. However, it was obvious that leprosy incidence was declining rapidly in the population (the project's interest shifted to tuberculosis, HIV, demographic surveillance, genetics and immunological studies), and thus leprosy ascertainment was left largely to the routine health services after 2000. Biopsies were no longer collected routinely and the diagnostic certainty assessment was carried out by experienced Leprosy Control Assistant staff. Given that the project was well known for leprosy work in the district since 1979, and the fact that project staff working throughout the district were experienced in leprosy diagnosis, it is likely that ascertainment remained reasonably high, but some cases may have been missed.

4.2 CONSORT flow chart

The Consort flow chart of the complete trial with reference to leprosy outcomes is presented as **Figure S-8**.

Figure S-8: CONSORT flow diagram of complete trial with reference to leprosy outcomes. Columns in bold refer to repeat BCG versus placebo comparison.

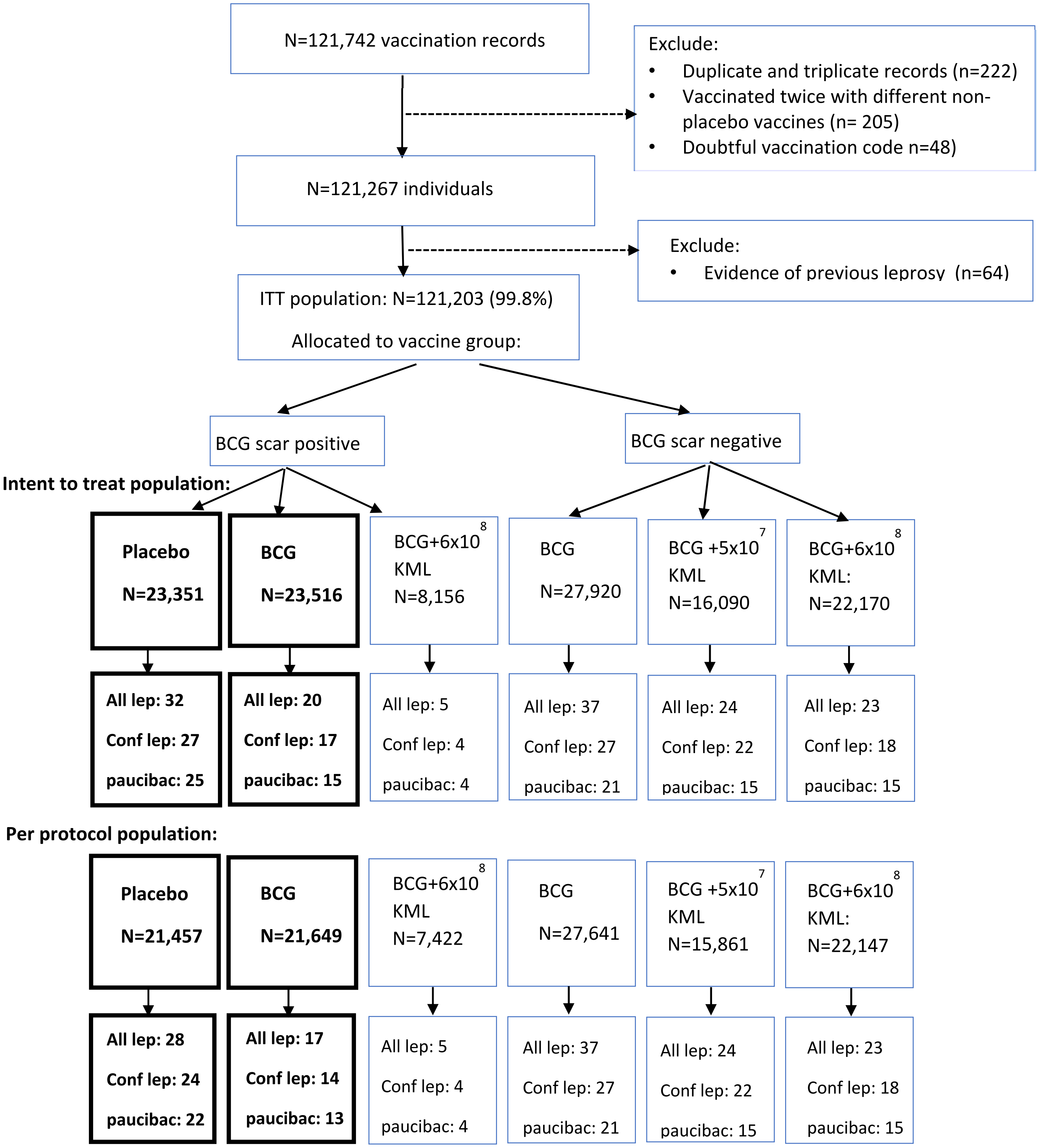

KML= killed *M. leprae*; Conf lep = confirmed leprosy; paucibac = paucibacillary

4.3 BCG vs placebo among BCG scar-positives

This is discussed in main paper, with reference to the ITT population. In the per-protocol analysis, which excluded 3761 individuals who received a vaccine inconsistent with their recorded scar status, results were very similar (**Figure S-9**).

Figure S-9: Odds ratios of leprosy associated with repeated BCG among scar positive individuals allocated either repeat BCG or placebo (per protocol population)

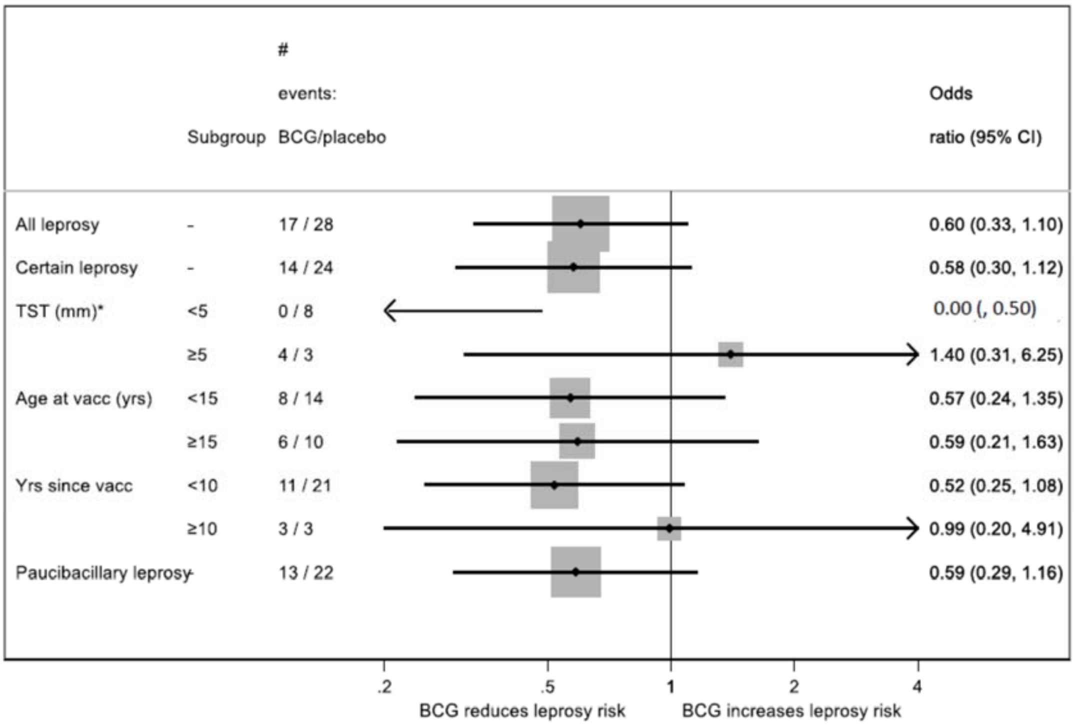

* Note: for subgroup <5mm: one-sided 95% CI calculated (Cornfield method)

In this new analysis with longer follow-up there were only 9 new cases, and no evidence that protection against leprosy induced by the second BCG vaccination lasted more than 10 years after the second BCG. This may reflect small numbers. On the other hand it may reflect waning of the vaccine's effect on the immune system, or masking of protection by cumulative exposure of the population to a variety of environmental mycobacteria which provide heterologous protection similar to that provided by BCG.[12] It was noted in 1995, and in this analysis, that protection against leprosy appeared to decline with age at vaccination, an observation which is consistent with the fact that protection was greatest among those with little or no tuberculin reactivity, and the fact that cumulative exposure to environmental as well as to tubercle mycobacteria rises with age. The evidence for strong protection (100 %, though based upon

only 8 cases) among individuals who were tuberculin negative at vaccination is consistent with this influence of exposure to other mycobacterial antigens.

4.4 BCG plus killed *M leprae* vaccines

Figures S-10 and S-11 present odds ratios for leprosy comparing BCG plus killed *M leprae* versus BCG alone, in scar negative and scar positive individuals respectively. Caution is required given the number of comparisons involved in these analyses. For neither group is there evidence that the addition of *M leprae* antigens influenced protection against leprosy. The only hint is a suggestion of greater protection among those given the combined BCG plus killed *M leprae* vaccine who were below 15 years of age at vaccination (OR=0.27; 0.07 - 1.03 among scar negatives; OR=0.33; 0.04-2.58 among scar positives). We note that there was no evidence of greater protection with the combined vaccine among those who were scar negative and tuberculin negative (<5 mm) at vaccination, which might have provided some rationale for protection among the young. The number of cases were small, and confidence intervals wide. **Table S-7** shows combined results of BCG plus killed *M leprae* versus BCG in scar negatives and scar positives (OR=1.11; 0.73-1.68) We conclude that there is no evidence that the addition of killed *M Leprae* antigens influenced protection imparted by BCG alone in this population.

Figure S-10: Odds ratios of leprosy comparing BCG+KML vs BCG among scar negative individuals (ITT).

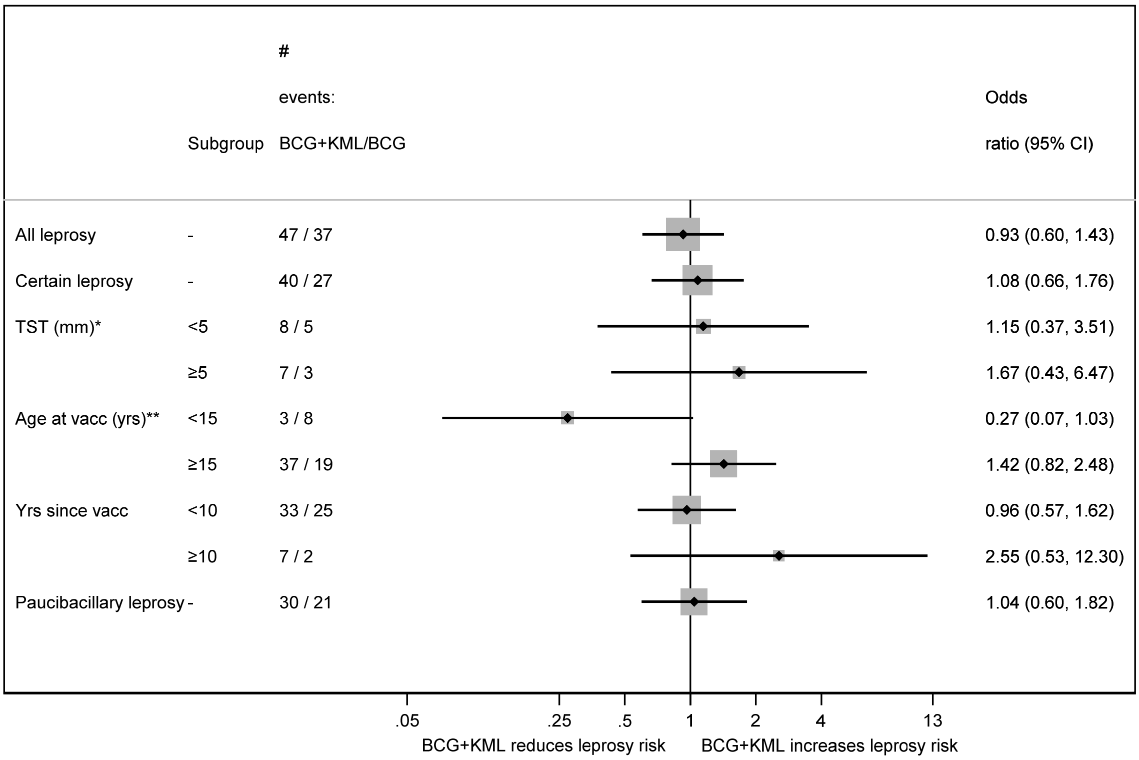

*interaction P-value 0.7; **interaction P-value 0.02

Figure S-11: Odds ratios of leprosy comparing BCG+KML vs BCG among scar positive individuals (ITT).

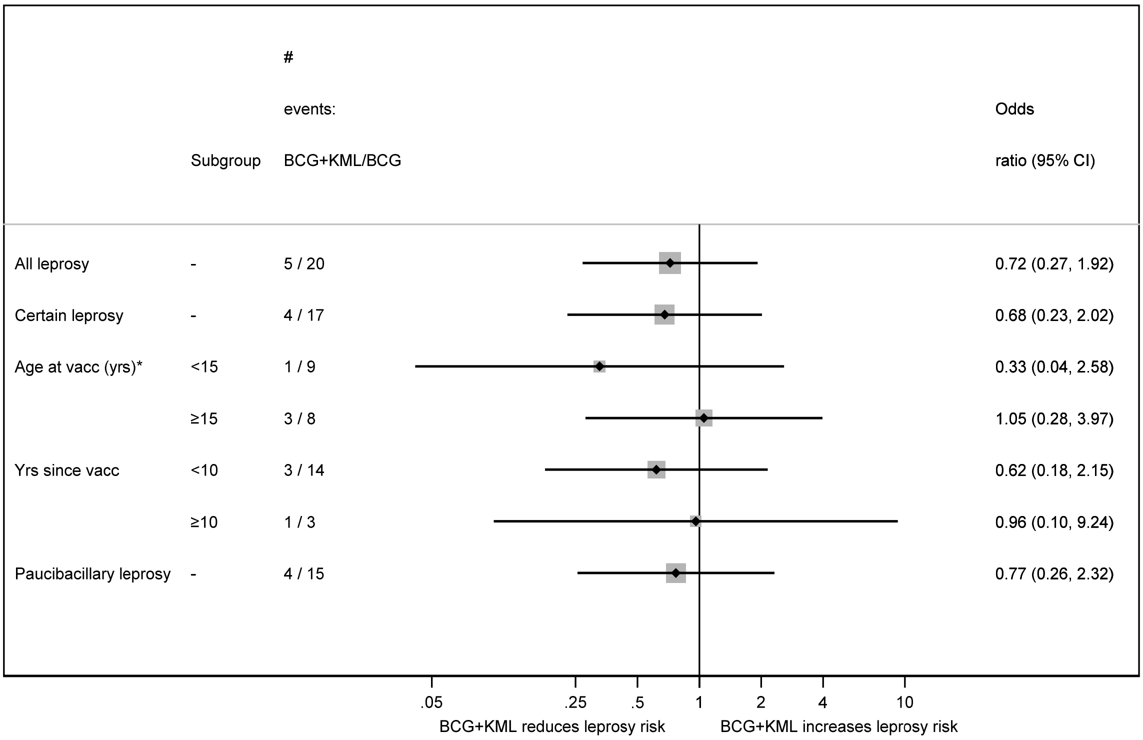

*interaction P-value 0.3

Table S-7: Odds ratios for certain leprosy combining comparisons between BCG plus killed *M leprae* versus BCG in both scar negatives and scar positives (ITT)

Vaccine	N	# certain leprosy	row%	OR	lower 95%	upper 95%
BCG+KML	46,416	44	0.09%	1.11	0.73	1.68
BCG	51,436	44	0.09%	1		

Given this conclusion, of no influence associated with the addition of killed *M leprae* antigens to BCG on protection against leprosy, we combine both BCG and BCG plus killed M leprae vaccines and compare them to placebo recipients among scar positive individuals in **Table S-8**. This adds four cases to the initial analysis of BCG versus placebo, providing once again an overall estimate of approximately 40 % protection by repeat BCG over the 30 years follow-up on this trial (OR= 0.57; 0.32 -1.01).

Table S-8: Odds ratios for certain leprosy combining BCG and BCG plus killed M leprae together, against placebo, in scar positives (ITT)

Vaccine	N	# certain leprosy	row%	OR	lower 95%	upper 95%
BCG+KML & BCG	31,672	21	0.07%	0.57	0.32	1.01
placebo	23,351	27	0.12%	1		

4.5 Discussion

In addition to the Karonga trial, two other trials were organised to evaluate the efficacy of BCG plus killed *M leprae* vaccine against leprosy, in Venezuela, and in South India. The Venezuela trial compared the combination vaccine against BCG alone among 29,113 contacts of leprosy patients (many of whom had already received a BCG vaccination). No effect on leprosy incidence was observed in this comparison.[14] The South India trial took place in a general population, where 171,400 individuals were randomised to receive one of five interventions: placebo, BCG, BCG + killed *M leprae,* "ICRC", or "Mw" (the latter two were killed preparations of different environmental mycobacterial species). Efficacy of BCG alone and of the BCG + killed *M leprae* vaccines were reported to be 34.1 % (95% CI 13.5 - 49.8), and 64 % (50.4 – 73.9), respectively There was no formal comparison between BCG+ killed *M leprae* and BCG alone, but these results imply a relative efficacy of the combined vaccine versus BCG alone of 45 %.[15,16] Neither trial was designed to evaluate protection against tuberculosis.

Once again we see examples of differing efficacy of mycobacterial vaccines between different populations. The immunological reasons for these differences continue to be debated, amid repeated calls for the experiences with vaccines against tuberculosis and leprosy to be considered together in order to guide research for the future.[17]

5. References:

1. Pönnighaus JM, Fine PEM, Bliss L, Sliney IJ, Bradley DJ, Rees RJW. The Lepra Evaluation Project, an epidemiological study of leprosy in Northern Malawi. I - Methods. Lepr Rev 1987; **58**: 359-375.

2. Pönnighaus JM, Fine PEM, Maine N, Bliss L, Kalambo M, Pönnighaus I. The Lepra Evaluation Project (LEP), an epidemiological study of leprosy in Northern Malawi. II. Prevalence rates. Lepr Rev 1988; **59**: 97-112.

3. Fine PEM, Pönnighaus JM, Burgess P, Clarkson JA, Draper CC. Seroepidemiological studies of leprosy in northern Malawi based on an enzyme-linked immuno-sorbent assay using synthetic glycoconjugate antigen. Int J Lepr 1988; **56**: 243-254.

4. Pönnighaus JM, Fine PEM, Sterne JAC, Bliss L, Wilson RJ, Malema SS, Kileta S. Incidence rates of leprosy in Karonga District, Northern Malawi: patterns by age, sex, BCG status and classification. Int J Lepr 1994; **61**: 10-23.

5. Pönnighaus JM, Fine PEM, Sterne JAC, Wilson RS, Msosa E, Gruer PJK, Jenkins PA, Lucas SB, Liomba G, Bliss L. Efficacy of BCG against leprosy and tuberculosis in northern Malawi. Lancet 1992; 339: 636-639.

6. Fine PEM, Pönnighaus JM. Background, design and prospects of the Karonga Prevention Trial, a leprosy vaccine trial in Northern Malawi. Transactions of the Royal Society of Tropical Medicine and Hygiene 1988; **82**: 810-817.

7. Fine PEM, Pönnighaus JM, Maine NM. The distribution and implications of BCG scars in northern Malawi. Bull WHO 1989; **67** (1): 35-42

8. Fine PEM, Pönnighaus JM, Maine N, Clarkson JA,Bliss L. Protective efficacy of BCG against leprosy in Northern Malawi. Lancet 1986; **ii**: 499-502.

9. Pönnighaus JM, Fine PEM, Bliss L, Gruer PJK, Kapira-Mwamondwe B, Msosa E, Rees RJW, Clayton D, Pike MC, Sterne JAC, Oxborrow SM. The Karonga Prevention Trial: a leprosy and tuberculosis vaccine trial in Northern Malawi: 1 - methods of the vaccination phase. Lepr Rev 1993; **64**: 338-356.

10. Karonga Prevention Trial Group. Randomised controlled trial of single BCG, repeated BCG, or combined BCG and killed *Mycobacterium leprae* vaccine for prevention of leprosy and tuberculosis in Malawi. Lancet 1996; **348**: 17-24.

11. Jahn A, Crampin AC, Glynn JR, Mwinuka V, Mwaiyeghele E, Mwafilaso J, Branson K, McGrath N, Fine PEM, Zaba B. Evaluation of a village-informant driven demographic surveillance system in Karonga, Northern Malawi. Demographic Research 2007; **16**: 219-247.

12. Fine PEM. Variation in protection by BCG: implications of and for heterologous immunity. Lancet 1995; **346**: 1339-1345.

13. Pönnighaus JM, Fine PEM, Bliss L. Certainty levels in the diagnosis of leprosy. Int J Lepr 1987; **55**: 454-462.

14. Convit J, Samson C, Zuniga M, et al. Immunoprophylactic trial with combined *Mycobacterium leprae*/BCG vaccine against leprosy: preliminary results. *Lancet* 1992; **339:** 446–50.

15. Gupte MD, Vallishayee RS, Anantharaman DS, Nagaraju B, Sreevatsa, Balasubramanyam S, de Britto RL, Elango N, Uthayakumaran N, Mahalingam VN, Lourdusamy G, Ramalingam A, Kannan S, Arokiasamy J. Comparative leprosy vaccine trial in South India. Indian J Lepr. 1998; 70(4): 369-388.

16. Gupte MD. South India immunoprophylaxis trial against leprosy: relevance of findings in the context of leprosy trends. Int J Leprosy 2001; **69** (suppl 2): S10-S13.

17. Coppola M, van den Eeden SJF, Robbins N, Wilson L, Franken KLMC, Adams LB, Gillis TP, Ottenhoff THM, Geluk A. Vaccines for leprosy and tuberculosis: opportunities for shared research, development and application. Frontiers in Immmunology 2018; 9:1-12.

6. Karonga Prevention Trial Group.

For the original trial

Coordinator—P E M Fine (London School of Hygiene and Tropical Medicine, London, UK).

Fieldwork directors—J M Pönnighaus (LEPRA, Chilumba, Malawi), D K Warndorff (LEPRA).

Group participants—Field medical officers: P J K Gruer, S Oxborrow,P D P Pharoah (LEPRA); histopathologists: S B Lucas (University College Hospital Medical School, London, UK), A C McDougall (Slade Hospital, Oxford, UK); mycobacteriologist: P A Jenkins (UKPHLS Mycobacterium Reference Laboratory, Cardiff, UK); senior paramedical staff: D Chavula, G Msiska, E Msosa, M Munthali, B Mwamondwe, D Ng'oma, P Nkhosa, H Phiri, M Simfukwa (LEPRA); senior interviewers: A Chihana, D Kawaonja, S Malema, V Mwinuka (LEPRA); senior laboratory staff: P Mkandawire, S Nyasulu, H Tegha (LEPRA); senior data managers: M Kalambo, S Kileta, M Simwaka (LEPRA); computing staff: L Bliss, J Saul (LSHTM); statisticians: N Maine, J A C Sterne (LSHTM); epidemiologist: J R Glynn (LSHTM); Clinical monitor: D Bell (Liverpool School of Tropical Medicine, Liverpool, UK); WHO monitors: D Clayton (MRC Biostatistics Unit, Cambridge), UK, M C Pike (ICRF Cancer Epidemiology Unit, Oxford, UK).

Diagnostic review team—I Cree (Institute of Ophthalmology, London, UK), K Desikan (MG Institute of Medical Sciences, India), M D Gupte (Indian Council of Medical Research, Avadi, India), R R Jacobson (USPHS Hansen's Disease Centre, Carville, Louisiana, USA), D S Nyangulu (Ministry of Health, Lilongwe, Malawi).

Data monitoring committee—D Clayton, J Darbyshire (MRC HIV Clinical Trials Unit, London, UK), M D Gupte, R Peto (ICRF/MRC/BHF Clinical Trials Service Unit, Oxford, UK).

Contributors to follow-up post 1995:

(excluding those who were part of the original trial group)

S Alghamdi, L Banda, K Branson, S Chaguluka, A Crampin, F Chilongo, R Chiumya, A Chiwaya, T Clark, R Dacombe, A Dimba, F Drobniewski, N French, S Geis, J Guerra-Assunção, R Houben, P Kahn, S Kasimba, M Kayange O Koole, K Kranzer, K Mallard, S Mboma, N McGrath, E McLean, R McNerney, B Mhango, P Mkandwire, J Mpunga, D Mulawa, K Munthali, L Munthali, M Munthali, H Mvula, E Mwaiyeghele, D Mwafulirwa, F Mwaungulu, J Mwaungulu, L Mwaungulu,, M Mwenibabu, P Mzumara, R Ndlovu, BM Ngwira, S Nyasulu, M Nyirenda. F Sibande, H Traore, M Yates
